# Advancing Cancer Prevention Practice Facilitation Work in Rural Primary Care During COVID-19

**DOI:** 10.13023/jah.0204.02

**Published:** 2020-09-01

**Authors:** Dannell Boatman, Susan Eason, Mary Ellen Conn, Summer Miller, Stephenie Kennedy-Rea

**Affiliations:** West Virginia University Cancer Institute

**Keywords:** Appalachia, COVID-19, cancer screening, practice facilitation, cancer, public health

## Abstract

COVID-19 and the response to slow the virus spread in West Virginia (WV), including a statewide stay-at-home order, presented challenges to rural primary care clinics on the frontlines. These challenges affected critical quality improvement work, including cancer screening services. In this commentary, the authors present the results of a survey of WV primary care practices that highlight potential long-term implications and identifies opportunities for practice facilitators to partner with rural primary care clinics to address them.

**Figure:**
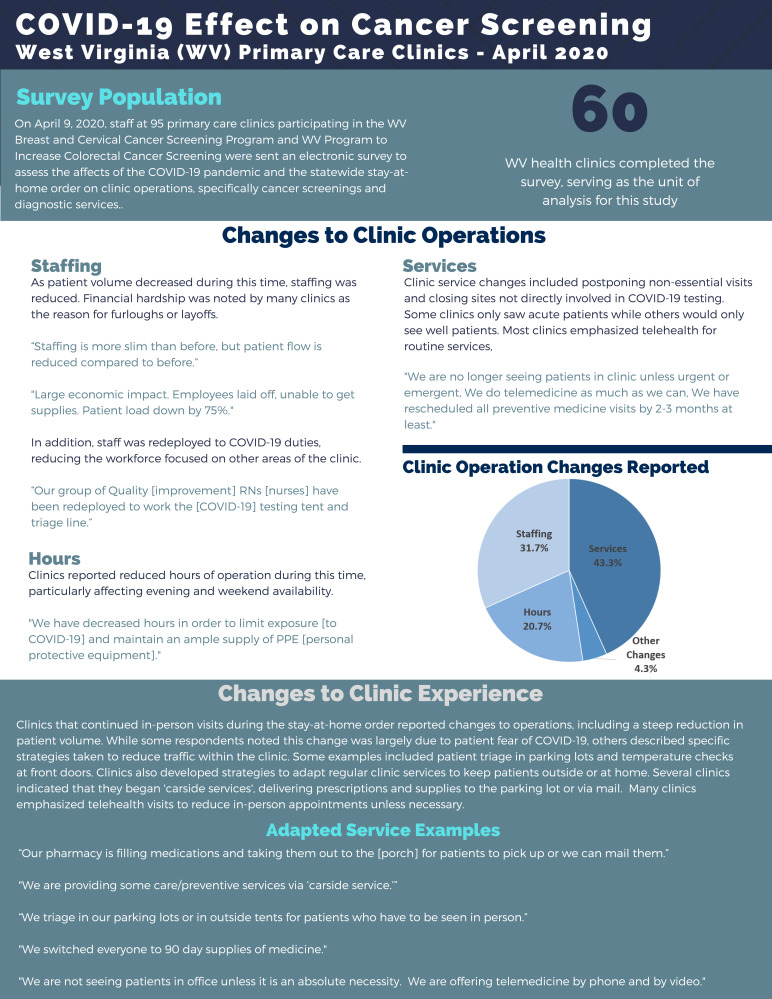


**Figure:**
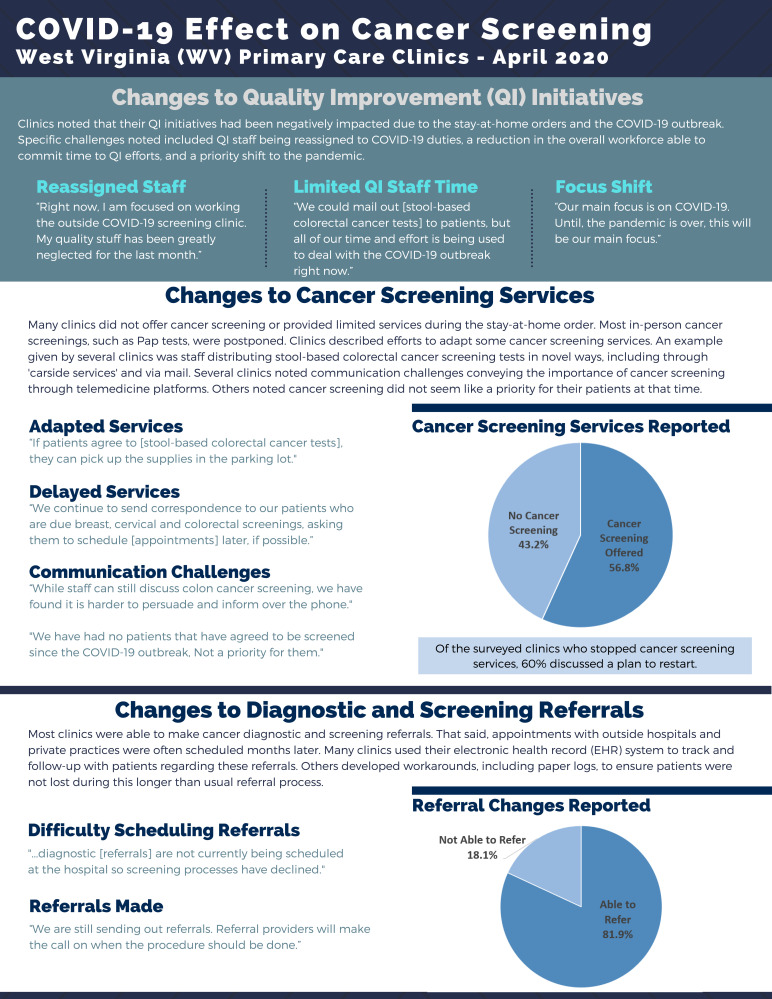


## INTRODUCTION

On March 24, 2020, West Virginia (WV) enacted a stay-at-home order^1^ to contain the spread of the novel coronavirus SARS-CoV-2 (COVID-19). While non-essential businesses across the state closed, primary care clinics began to adjust to a new reality. For public health professionals who partner with primary care clinics on quality improvement initiatives, in our case cancer prevention, this marked the start of a dramatic change in practice facilitation work. To assess these changes, we surveyed our partner WV primary care clinics in early April to gauge the effect of the stay-at-home orders and COVID-19. While survey results highlighted challenges for quality improvement initiatives, opportunities to adapt were also identified. Primary themes identified from this survey are presented in this commentary and recommendations to advance cancer prevention practice facilitation in this evolving landscape are suggested.

## CLINIC OPERATION CHANGES

Our team found that COVID-19 and the statewide stay-at-home order affected primary care clinic operations, leading to changes in service, staffing, and hours. Many clinics transitioned from in-person visits to telehealth and reported a learning curve for both patients and staff. Staffing changes emerged due to reassignment of duties and furloughs. Survey participants noted that patient volume diminished due to the stay-at-home order, service delivery changes, and patient fears of COVID-19. Additionally, clinic hours were cut to reduce the amount of personal protective equipment (PPE) used. Clinics reported difficulty sourcing PPE, in line with challenges experienced across the country.^2^

## CANCER SCREENING CHANGES

Based on the survey results, we found that operational changes may have affected quality-improvement efforts, including cancer screening. Staff members charged with running these initiatives were often furloughed or reassigned to COVID-19 efforts. In addition, not all cancer screenings were offered. Referrals for diagnostic testing were difficult for some clinics to schedule. Most clinics indicated that they were able to make referrals, but the appointments were scheduled months later. Survey participants reported efforts to adapt their services, like distributing stool-based colorectal cancer screening test kits in parking lots. That said, several survey participants noted communication challenges, such as difficulty effectively encouraging cancer screening over the phone.

## PRACTICE IMPLICATIONS

The WV stay-at-home order ended on May 4, 2020.^3^ Several clinic partners reported to our team an increase in patient volume at this time but indicted that challenges identified in early April remained.

Clinic partners noted that patient fear of COVID-19 has not diminished, particularly for those in at-risk categories. Survey participants noted that with this fear, it became more difficult to encourage patient compliance with cancer screening. This presents a challenge with patient engagement. It also offers an opportunity for those partnering with primary care clinics to work with them to adapt evidence-based interventions that can reach patients in new ways, like mailed stool-based colorectal cancer screening tests.^4^ It also provides the opportunity to work with primary care clinics on messaging that emphasizes the importance of cancer screening and how it can be done safely in-clinic.

Survey respondents indicated that COVID-19 spurred many primary care clinics to offer telehealth services for the first time. While this makes accessing primary care providers easier for some patients, survey participants also suggested that it presented obstacles related to the communication and delivery of cancer prevention services. New strategies to engage patients on this service delivery platform are needed as the health care landscape shifts during COVID-19 and beyond.

Clinic partners indicated that staff furloughs and layoffs significantly affected primary care clinic operations. There is no indication when these numbers will return to pre-COVID-19 levels. To that end, the workforce may not be available to engage in quality improvement initiatives at the same rate as before the pandemic. Further, staff reassignment to COVID-19 efforts may continue and affect various quality improvement projects. Public health professionals working with primary care clinics should offer understanding to partners. Exhibiting flexibility and increasing innovation can strengthen relationships in the short-term, leading to successful long-term partnerships.

## CONCLUSION

Professionals who work with primary care clinics on quality improvement initiatives, such as cancer prevention, need to remain cognizant of the challenges these partners are experiencing due to COVID-19. There are opportunities to continue to engage with primary care clinics in novel ways to overcome these trials and strengthen partnerships.
